# Quantifying Plant-Borne Carbon Assimilation by Root-Associating Bacteria

**DOI:** 10.3390/microorganisms8050700

**Published:** 2020-05-10

**Authors:** Spenser Waller, Stacy L. Wilder, Michael J. Schueller, Alexandra B. Housh, Richard A. Ferrieri

**Affiliations:** 1 School of Natural Resources, University of Missouri, Columbia, MO 65211, USA; sgwxhv@mail.missouri.edu; 2 Missouri Research Reactor Center, University of Missouri, Columbia, MO 65211, USA; wildersl@missouri.edu (S.L.W.); schuellerm@missouri.edu (M.J.S.); afbkhn@mail.missouri.edu (A.B.H.); 3 Chemistry Department, University of Missouri, Columbia, MO 65211, USA; 4 Division of Plant Sciences, Interdisciplinary Plant Group, University of Missouri, Columbia, MO 65211, USA

**Keywords:** *Herbaspirillum seropedicae*, green fluorescence reporting, endophytic rhizobacteria, maize roots, carbon-11, plant-borne carbon

## Abstract

*Herbaspirillum seropedicae* is a rhizobacteria that occupies a specialized ecological niche in agriculture. As an endophyte and prolific grass root colonizer it has the potential to promote plant growth, enhancing crop yield in many cereal crops. While the mechanisms for plant growth promotion are controversial, the one irrefutable fact is these microorganisms rely heavily on plant-borne carbon as their main energy source in support of their biological functions. Unfortunately, the tools and technology enabling researchers to trace carbon exchange between plants and the microorganisms associating with them has been limiting. Here, we demonstrate that radioactive ^11^CO_2_ administered to intact maize leaves with translocation of ^11^C-photosynthates to roots can provide a ‘traceable’ source of carbon whose assimilation by microbial organisms can be quantified with enormous sensitivity. Fluorescence root imaging of RAM10, a green fluorescent protein (GFP) reporting strain of *H. seropedicae*, was used to identify regions of high microbial colonization. Microbes were mechanically removed from these regions via sonication in saline solution and extracts were subjected to fluorescence measurement and gamma counting to correlate carbon-11 atoms with numbers of colony forming units. The method has potential to translate to other microorganisms provided they possess an optical reporting trait.

## 1. Introduction

Grain crops, such as maize, provide an estimated 30% of the food calories to more than 4.5 billion people in developing countries. Large amounts of nitrogen (N) fertilizer are required to obtain a maximum yield for maize, but maize use of this fertilizer is less than 50% efficient leaving a significant portion to environmental runoff [1,2]. Over the years, increases in crop yields can be attributable, in part, to an ever-increasing use of N fertilizer, and these actions have concomitant negative impacts on climate and water resources [3]. A major challenge for sustainable agriculture is how to deliver N to the plant to maintain high yield, while preventing the negative consequences of N fertilizer addition [4,5,6]. One of the most promising agro-ecological approaches that could enable the reduction of N fertilizer application, while maintaining crop productivity, is to better exploit the beneficial effects of soil microbiota and especially biologically N_2_-fixing (BNF) bacteria.

A variety of BNF bacteria are commonly present in the plant rhizosphere that can establish close associations with roots, colonizing the roots either epiphytically or endophytically [7,8,9]. Indeed, plant growth promoting bacteria (PGPB) can reach dense concentrations (e.g., 10^8^/g) in roots without inducing a noticeable plant defense response [10,11,12]. Previous studies showed that PGPB commonly impact root architecture and plant health, attributing these effects to such things as BNF, stress tolerance enhancement, production of phytohormones, enhancement of nutrient acquisition, and protection against pathogens and pests [13,14]. The growth of root-associating microbes is usually carbon-limited, so the sugars, amino acids, and organic acids that plants deposit into the rhizosphere represent a valuable nutrition source [15]. BNF is a particularly costly microbial function placing additional demands on microbial carbon resources.

Although recent progress has been made in our understanding of atmospheric carbon fluxes belowground, knowledge is still scarce with respect to how much plant-borne carbon is assimilated by specific microorganisms in plant-soil systems [16]. This is due to the lack of tools and technology capable of quantifying these fluxes. To the best of our knowledge, only a few applications have been published that used ^13^C stable isotope probing to track plant-derived carbon fluxes into microbial nucleic acids [17,18] and other biomarkers [19], as well as into fungal-specific metabolism [20,21] that opened a window to understanding the flux of carbon through plant-associated communities belowground.

**Figure 1:**
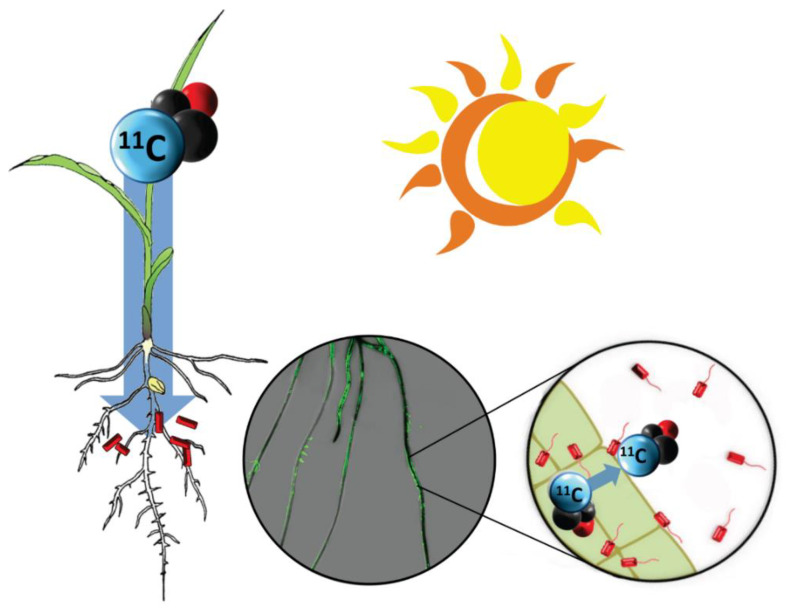
Concept of plant-borne carbon tracing to root-associating rhizobacteria. Atmospheric carbon is traced from plant fixation of radioactive ^11^CO_2_ through root translocation as complex ^11^C-photosynthates. There, that carbon source is assimilated by root-associating microorganisms. The center insert shows an overlay of images from ^11^C-radiography (dark black regions reflect high levels of ^11^C activity) and a green fluorescence image arising from RAM10, a green fluorescent protein (GFP) reporting strain of *H. seropedicae* bacteria. Regions of high root colonization seem to draw more ^11^C.

Here we describe a novel approach using radioactive ^11^CO_2_ to track the incorporation of atmospheric carbon into plant carbohydrates with subsequent translocation of complex ^11^C-photosynthates to roots where they are assimilated by root-associating bacteria (Figure 1). Unlike stable isotope probing, this nuclear-based method is quantifiable providing an absolute measurement of microbial carbon assimilation of plant-derived products. It is also enormously sensitive owing to the short-lived nature for radioactive decay of this isotope (^11^C, t_½_ 20.4 m), which allows measurement using gamma counting of minuscule quantities of tracer in very small numbers of bacteria cells.

## 2. Materials and Methods

### 2.1. Plant Growth

Maize kernels from Elk Mound Seed Co. (Hybrid 8100, Elk Mound, WI, USA) were surface sterilized in 0.6% bleach solution for 15 min, rinsed well in sterile water, and dark germinated at room temperature for two days on sterilized paper towels wetted with sterile water in a petri dish and then allowed to grow for an additional two days before inoculation with bacteria. Seedlings were transplanted into plastic seed germination pouches (Phytotc, Inc.; Shah Alam, Selangor, Malaysia), wetted with sterile Hoagland’s basal salt solution, and allowed to grow for approximately one week. At this time they were transplanted into conical plastic pots filled with Turface™, an expanded clay medium. Nutrient was introduced as Hoagland’s solution every three days. Growth conditions consisted of 12-h photoperiods, 500 μmol m^−2^ s^−1^ light intensity, and temperatures of 25/20 °C (light/dark) with humidity at 70–80%. Plants were studied after three weeks of growth.

### 2.2. Bacteria Growth and Root Inoculation

RAM10, a green fluorescence reporting strain of *Herbaspirillum seropedicae,* was grown in liquid NFbHP-malate medium following published procedures [13]. The bacterial cultures were grown in 25 mL flasks in a shaking incubator set to 30 °C and 130 rpm until OD_600_ = 1.0 (optical density at 600 nm, corresponding to 10^8^ cells mL^−1^) was reached. Cultures were then washed with sterile water and diluted to a 1 mL suspended solution of approximately 10^6^ to 10^8^ colony forming units (CFU mL^−1^). Root inoculation involved adding 1 mL of inoculum to a petri dish of 10 germinated maize seedlings and gently rocking the dish in the shaking incubator for two hours.

### 2.3. Production and Administration of Radioactive ^11^CO_2_

^11^CO_2_ (t_½_ 20.4 min) was produced on the GE PETrace Cyclotron located at the Missouri Research Reactor Center using high-pressure research grade N_2_ gas target irradiated with a 16.4 MeV proton beam to generate ^11^C via ^14^N(p,α)^11^C nuclear transformation [22,23]. The ^11^CO_2_ was trapped on a molecular sieve, desorbed, and quickly released into an air stream at 200 mL min^−1^ as a discrete pulse for labeling a leaf affixed within a 5 × 10 cm lighted (560 μmol m^−2^ s^−1^) leaf cell to ensure a steady level of fixation. The load leaf affixed within the cell was pulse-fed ^11^CO_2_ for 1 min and then chased with normal air for the duration of exposure. A PIN diode radiation detector (Carroll Ramsey Associates, Berkeley, CA, USA) attached to the bottom of the leaf cell enabled continuous measurement of radioactivity levels within the cell during the initial pulse and in the minutes directly following to give information on ^11^CO_2_ fixation and leaf export of ^11^C-photosynthates [24].

Translocation of ^11^C-photosynthates to roots was allowed to occur for one hour after which plant tissues (shoots and roots) were collected and quantified for ^11^C-activity using a gamma counter. However, roots were first rinsed in de-ionized water to remove ^11^C-labeled root exudates. Turface™ medium was also counted for ^11^C-activity and combined with the wash activity as ^11^C-root exudates.

### 2.4. Autoradiography

**Figure 2:**
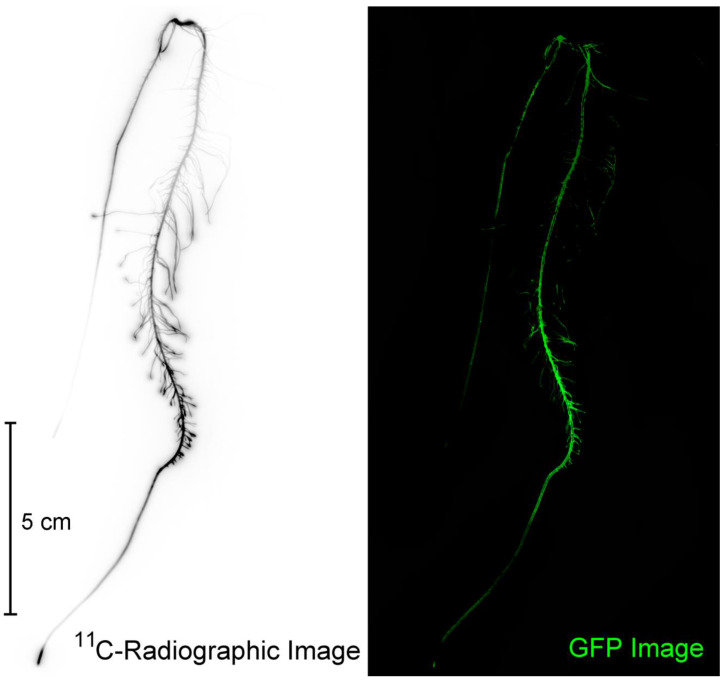
A comparison of the same root subjected to ^11^C-radiographic imaging and GFP imaging on the Typhoon 9000. Using nuclear-based and optical-based imaging, we were able to identify regions of high ^11^C-activity and bacteria colonization.

Subsequent to ^11^C exposures, plants were harvested, roots were laid out, and radiographic images acquired by exposing phosphor plate films for on average 10 min. Phosphor plates were read using a Typhoon 9000 imager (Typhoon^TM^ FLA 9000, GE Healthcare, Piscataway, NJ, USA). Images were used qualitatively only for determining spatial patterning of ^11^C activity (Figure 2).

### 2.5. Fluorescence Imaging

**Figure 3:**
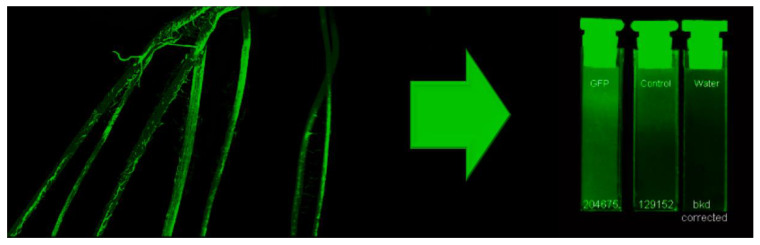
Workflow showing green fluorescence imaging to identify regions of root tissues with highest levels of root colonization by RAM10, *H. seropedicae,* bacteria. Isolated 4-cm portions of root were subjected to sonication in saline solution to mechanically remove bacteria. The extract was re-imaged using the GFP signature of the microorganisms to quantify bacteria content in the liquid extract. Background corrections were made in each set of measurements using a deionized water sample. Non-inoculated control roots exhibited a trace amount of fluorescence signal attributable to naturally fluorescent plant metabolites.

The Typhoon 9000 imager was also used to perform low resolution (100 μm) fluorescence imaging using blue laser excitation light (473 nm wavelength) and green light emission (ThorLabs, Inc., Newton, NJ, USA; Filter No. MF525-39; 35 nm bandwidth centered at 525 nm) of the entire root mass. Roots were rinsed in deionized water before they were staged on a fluoroplate imaging cassette. This low resolution image enabled identification of regions where there was a high level of microbial colonization by RAM10 (Figure 2). After identifying regions of high microbial density, 4-cm pieces were cut from the total root mass, weighed, and counted for ^11^C-activity before end-capping each piece in paraffin wax. This action ensured that root metabolites that were radiolabeled and/or possessed a natural fluorescence signature would not ‘leak’ from the cut ends during sonication contaminating the extract. The extract was transferred to quartz optical quality cuvettes (Figure 3) and imaged for its fluorescence signature using the Typhoon 9000. Non-inoculated control roots were treated in the same fashion and used to correct microbial samples for natural fluorescence plant metabolites. The cuvettes were also counted for ^11^C-activity. Cuvette fluorescence was quantified using ImageQuant TL 7.0 software (Cytiva Life Sceinces; Marlborough, MA, USA).

During development of the method, we subjected several root pieces to isolated green fluorescent protein (GFP) imaging to monitor the effect of sequential removal of bacteria during serial sonication in saline solution on root fluorescence and the ^11^C activity signatures (Figure S1).

A subset of colonized roots were mounted in optimal cutting temperature compound (OCT), sectioned on a cryotome (Fisher Scientific Inc., Hampton, NH, USA) to 100 μm thickness, and placed on quartz microscope slides for further GFP imaging giving a measure of the extent of internal colonization versus surface colonization. ImageJ software was used to analyze and quantify GFP signatures on the internal regions of the root versus the root surface. As an endophyte, *H. seropedicae* is capable of colonizing both regions of the root.

### 2.6. Bacteria Quantification—Drop Plate Assay

Bacterial quantifications were performed concurrently with bacteria tracer studies. Plants were harvested from the growth media and rinsed in deionized water. Two separate ~3 cm sections of root were taken from each plant and weighed (approximately 100–300 mg total). The sample was shaken using a Retsch ball mill grinder (Verder Scientific, Inc., Newtown, PA, USA) for approximately 5 min to macerate the section. Then 1 mL of sterile 1% saline solution was added and vortexed with the sample. Five serial dilutions were performed: the first with 100 µL of the ground extract into 900 µL of 1% saline solution and each subsequent dilution being 100 µL of the previous dilution into 900 µL of 1% saline solution. Each serial dilution was plated in triplicate using 10 μL drops onto agar plates fortified with malate growth media and incubated at 30 °C for 48–72 h before counting. ImageQuant software was used in colony counting mode setting to quantify GFP exhibiting colonies measured on the Typhoon 9000 imager.

### 2.7. Root Sonication 

End-capped roots were sonicated for 2 min in 1% saline solution at 20 °C using 50% power (Branson Bransonic 32; Sigma-Aldrich Corp. St. Louis, MO, USA). This procedure followed best practices from prior published work [25]. The liquid extract was transferred to optical cuvettes for fluorescence measurement. Additionally, roots were re-imaged on the Typhoon 9000 for remaining tissue fluorescence after sonication (see Supplementary Figure S1). For serial sonication tests, this process was repeated six times.

## 3. Results and Discussion

**Figure 4:**
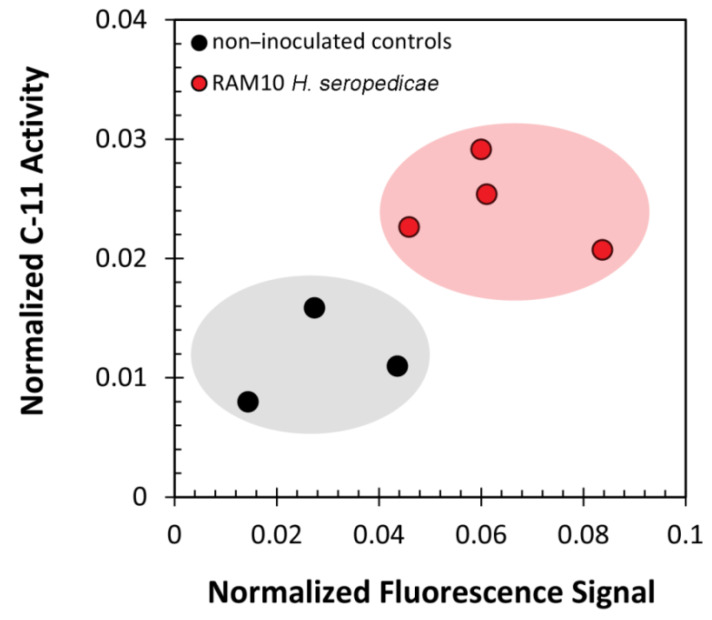
Scatter plot showing cuvette ^11^C activity plotted against its fluorescence signature. Data was normalized to a fixed mass of root tissue used to generate each sample. Gray and pink zones were drawn to accentuate the trends between inoculated and non-inoculated maize.

The plot of ^11^C-activity against the fluorescence signature from root sonication washes (Figure 4) exhibited a clear clustering of data around the presence or absence of RAM10 *H. seropedicae*. In general, the presence of *H. seropedicae* resulted in 2.5-fold higher ^11^C activity values and 2.2-fold higher fluorescence values than non-inoculated control roots. We note that non-inoculated control roots exhibited a small amount of both ^11^C-activity and fluorescence. Plants possess a natural autofluorescence signature attributable to the numerous secondary metabolites that contain fluorophores [26]. Hence, the small level of activity and fluorescence may reflect some amount of root exudates that were not removed during the initial root rinsing in deionized water.

**Figure 5:**
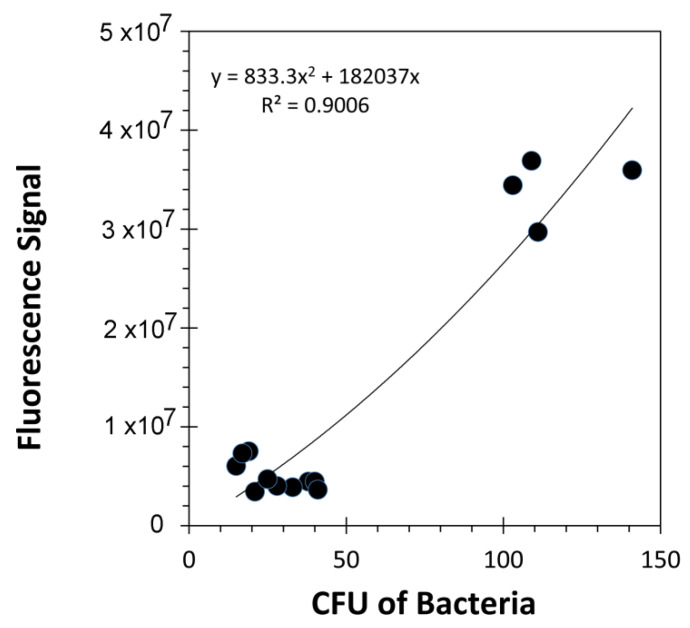
Drop plate assays enabled correlating RAM10 fluorescence with colony forming units from liquid cultures (CFUs).

**Figure 6:**
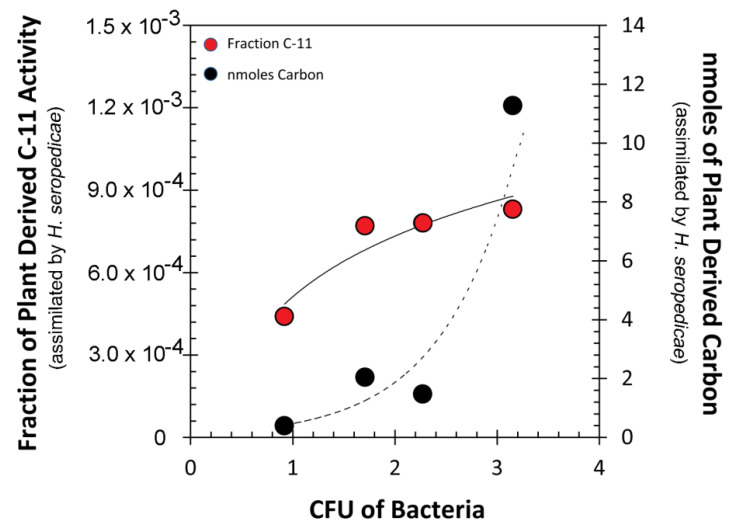
Representation of ^11^C assimilated in bacteria as a function of bacterial concentration (CFU) calculated from plated samples of tissue extract. The left-side axis presents plant-borne carbon assimilation as the fraction of ^11^C in the entire plant. Activity data was normalized for different ^11^C-allocation values due to variable root mass. The right-side panel presents a different perspective where the absolute activity values (in disintegrations per minute) were re-calculated in molar mass units. Raw data that generated this figure and the calculations used to arrive at final values can be found in Supplementary Table S1.

**Figure 7:**
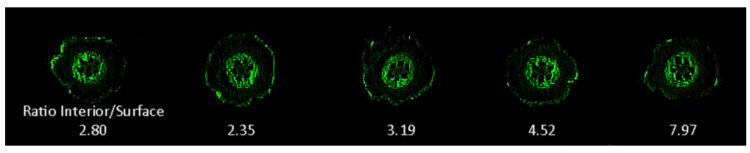
GFP images of 100 μm thick fresh root. GFP root slices revealed a higher level of internal root colonization within the vascular core than the surface of the root.

**Figure 8:**
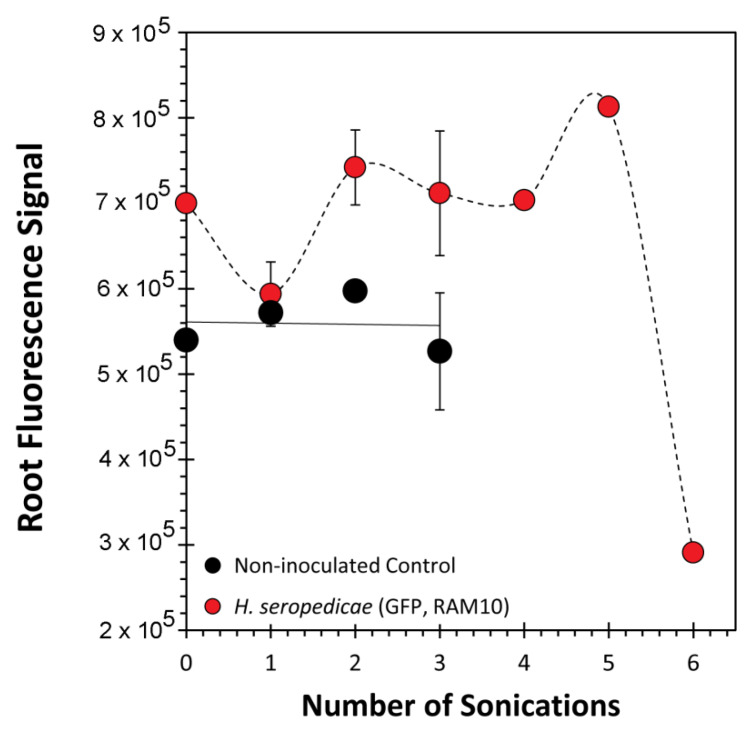
Fluorescence measurements of serially sonicated paraffin end-capped roots. For 0–3 sonications, data represent an average value (*n* = 12) ± SE. Sonication 4–6 represents an average of two root samples.

The fluorescence signature from samples containing RAM10 *H. seropedicae* were correlated to colony forming units (CFUs) of bacteria using data from standard drop plate assays of RAM10 liquid cultures where bacteria counts were conducted using the GFP signature (Figure 5). Trend analysis of data resulted in a non-linear best fit to a polynomial function. This function was used to re-calculate fluorescence values from Figure 4 as CFUs presented in Figure 6 on the x-axis. Normalized ^11^C- activity was calculated to reflect the fraction of ^11^C-activity within the entire plant that was assimilated by the small sample of bacteria surveyed in the analysis (left-side axis, Figure 6). ^11^C-activity values are presented in units of molar carbon mass (right-side axis, Figure 6). Both perspectives for viewing bacteria carbon assimilation showed increasing values with increasing CFUs of bacteria in the surveyed sample. GFP imaging of root sections with analysis using ImageJ software revealed that, on average, the ratio of internal colonization to surface colonization was 4.21 ± 1.01 (Figure 7). Because *H. seropedicae* is an endophyte, it was determined whether the sonication process in saline solution could draw internally colonized bacteria to the root surface. In this exercise we subjected several paraffin-capped root pieces to a series of sonications in saline solution measuring the root fluorescence signature at each step in the process (Figure 8). Results showed an interesting oscillatory pattern in the root fluorescence signature from roots that were inoculated with RAM10 *H. seropedicae*. This behavior was not observed for non-inoculated control roots. These oscillations may be indicative of a physical process where internally colonized bacteria are drawn to the root surface by the sonication in saline solution where their GFP signature would be less attenuated by the root tissue manifesting in a brighter fluorescence signature. With the next sonication, these bacteria are removed, lowering root brightness. This process could continue until the population of internally colonized bacteria is depleted.

**Figure 9:**
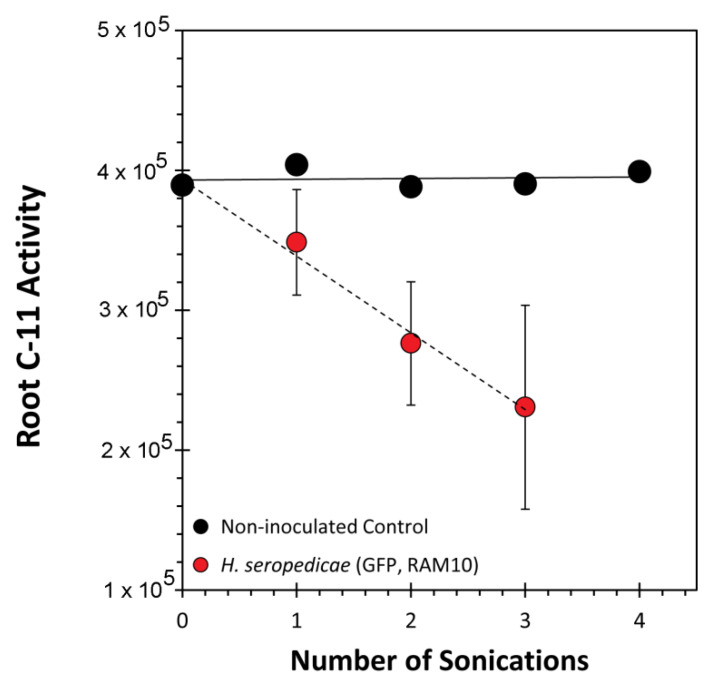
^11^C-Activity measurements of serially sonicated paraffin end-capped roots. Data from *H. seropedicae* inoculated roots represent average values (*n* = 3) ± SE. Data from the non-inoculated root represents a single sample. All ^11^C-activity data was decay corrected back to a common time point.

In parallel studies, the exercise was repeated for ^11^C activity measurements by applying serial sonications to selected roots after plants were exposed to ^11^C (Figure 9). RAM10 *H. seropedicae* inoculated roots had a linear decline in the level of ^11^C-activity with each sonication. We noted that unlike the fluorescence measurements, measurement of ^11^C-activity by gamma counting will not be subjected to the same level of tissue attenuation phenomenon. Hence, the linear decline in ^11^C-activity is due to the continual removal of bacteria as it is drawn to the surface. This interpretation was validated when a non-inoculated root was subjected to the same test. Here, no change in ^11^C-activity tells us that root cellular ^11^C-activity is not escaping in the sonication process.

## 4. Conclusions

Here we report a novel approach using radioactive ^11^C to trace the flow of atmospheric carbon belowground where it can be assimilated by root-associating microorganisms. Unlike past techniques that have relied on ^13^C stable isotope probing, our method is quantifiable in absolute units of carbon and is highly sensitive to the responses of even small colonies of microorganisms. Of course, a caveat of the method is that the microorganisms must possess an optical reporter.

## Supplementary Materials

The following are available online at https://www.mdpi.com/2076-2607/8/5/700/s1, Figure S1: Green fluorescent images of root pieces before and after sonication in saline solution.; Table S1: Raw radioactivity count data and fluorescence data from Figure 6.
