# Supplementary material for: Quantifying Plant-Borne Carbon Assimilation by Root-Associating Bacteria
Source: Microorganisms. 2020 May 10;8(5):700. doi: 10.3390/microorganisms8050700 (PMC7285072; doi:10.3390/microorganisms8050700)
Supplement: Supplementary file 1 [file microorganisms-08-00700-s001.pdf]

microorganisms

Supplementary File

Communication

Quantifying plant-borne carbon assimilation by root-associating bacteria

Spenser Waller[1], Stacy L. Wilder[2], Michael J. Schueller[2, 3], Alexandra B. Housh[2, 3] and Richard A. Ferrieri[2, 3, 4, *]

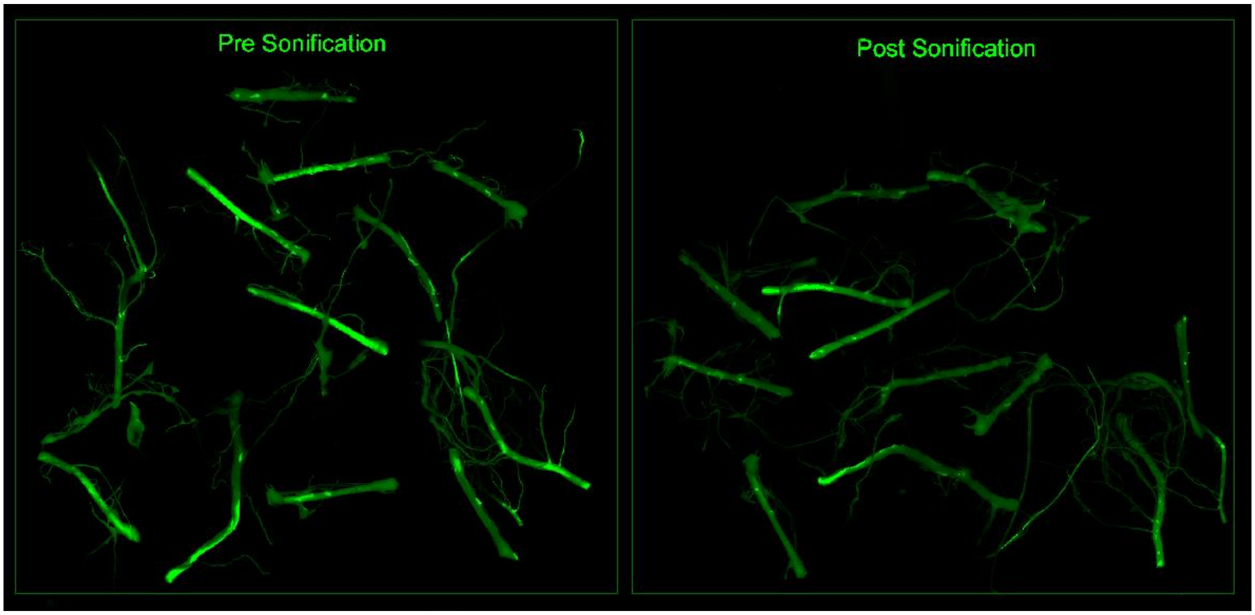

Fig. S.1. Raw radioactivity count data and fluorescence data from Figure 6. Root pieces were subjected to isolated green fluorescent protein (GFP) imaging to monitor the effect of sequential removal of bacteria during serial sonication in saline solution on root fluorescence.

microorganisms MDPI

Table S.1. Raw radioactivity count data and fluorescence data from Figure 6.

Study No.	Sample Counted	delta T (min)	Radioactivity (cpm) *	Decay Corrected Activity (cpm) *	Efficiency Corrected (dpm) **	Fraction Total Plant C-11 Activity	Number of C-11 Atoms	nmoles of Carbon	Cuvette Fluorescence	CFU
	Cuvette	146	39729	5663414	5663414	0.0769	6.79E+15	11.27	858647	3.15
	Shoots	257	34292	212213354	3021918163	41.0586				
1	Load Leaf	274	16237	179014385	2549164849	34.6354				
	Root Wash	187	559	320824	4568541	0.0621				
	Turface Medium	202	105	100692	1433854	0.0195				
	Roots	241	33792	121435174	1729236879	23.4950				
				**Total Plant Activity**	**7360006550**	**100**				
	Cuvette	104	29914	1023791	1023791	0.04	2.36E+14	0.39	250105	0.92
	Shoots	291	15517	304789720	3340205606	19.85				
2	Load Leaf	304	134950	1929926567	4601862151	68.01				
	Root Wash	104	14703	503203	7165604	0.03				
	Turface Medium	100	13067	390391	5559168	0.03				
	Roots	293	8797	184941180	2633562402	12.04				
				**Total Plant Activity**	**21870364184**	**100**				
	Cuvette	158	921	197366	197366	0.0831	1.23E+15	2.04	465015	1.71
	Shoots	208	37717	44178188	629097392	27.9328				
3	Load Leaf	304	24856	85377454	1215774942	53.9821				
	Root Wash	87	1335	25646	365194	0.0162				
	Turface Medium	113	24	1115	15880	0.0007				
	Roots	180	62867	28444988	405056633	17.9851				
				**Total Plant Activity**	**2252180895**	**100**				
	Cuvette	124	10949	739224	739224	0.0781	8.86E+14	1.47	618505	2.27
	Shoots	260	57533	394238619	5613957937	62.5394				
4	Load Leaf	262	26085	191501842	2726845101	30.3770				
	Root Wash	94	10290	250738	3570507	0.0398				
	Turface Medium	111	4274	185543	2642129	0.0294				
	Roots	249	9272	43725012	622644172	6.9363				
				**Total Plant Activity**	**8976667050**	**100**				

* cpm defined as radioactivity counts per minute.
** dpm defined as radioactivity disintegrations per minute.

Raw data is shown in Table S.1 reflecting the nature of applied calculations. Carbon-11 activity was counted on a gamma counter giving counts per minute (cpm). Samples included the cuvette which contained the sonication solution, the load leaf which was initially exposed to $^{11}CO_2$, the distal plant tissues where all ^{11}C-photosynthate translocated to and the root wash solution and Turface™ growth medium accounting for ^{11}C-exudates. Altogether, all components counted comprised the total amount of ^{11}C-activity fixed by the plant at the beginning of the study. The delta T values reflect the elapsed time in minutes from the end-of-bombardment (EOB) when the radioactivity was first produced on the cyclotron to the time when the sample was counted. Raw radioactivity counts were decay corrected back to EOB using the following equation:

$$A_o = A_T * \exp(\lambda T)$$

where A_o is the calculated decay corrected radioactivity at T_o or EOB, A_T is the measured radioactivity at time T, λ is the decay constant equal to $(\ln 2/t_{½})$ where $t_{½}$ is the half-life for ^{11}C equal to 20.4 min., and T is the elapse time from EOB to when the sample was counted.

After decay correction, data was corrected for intrinsic detector efficiency and geometry efficiency enabling us to convert counts per minute (cpm) to disintegrations per minute (dpm). All the samples were summed providing a total plant ^{11}C-activity value that was used to calculate

microbial ^{11}C-assimilation as the fraction of total ^{11}C-activity. Disintegrations per minute (dpm) can be related to theoretical number of ^{11}C atoms by the relationship:

$$2.22 \times 10^{12} \text{ dpm } = 1 \text{ Curie (Ci) of radioactivity (or } 3.7 \times 10^{10} \text{ Becquerel (Bq) in SI units)}$$

1 Curie of radioactivity is defined as the amount of radioactivity given off by 1 gm amount of ^{226}Ra. Using this relationship the number of ^{11}C atoms can be calculated and converted to molar mass units using Avogadro's number (6.023×10^{23} atoms/mole). Cuvette fluorescence values were converted to CFUs using the relationship from Fig. 5:

$$y = 833.3\, x^2 + 182037\, x$$

where y represents the experimentally measured cuvette fluorescence corrected for background fluorescence obtained from a deionized water sample and x is the calculated colony forming units (CFU) of bacteria.
